# Simulating Spinal Border Cells and Cerebellar Granule Cells under Locomotion – A Case Study of Spinocerebellar Information Processing

**DOI:** 10.1371/journal.pone.0107793

**Published:** 2014-09-16

**Authors:** Anton Spanne, Pontus Geborek, Fredrik Bengtsson, Henrik Jörntell

**Affiliations:** Neural Basis of Sensorimotor Control, Department of Experimental Medical Science, Lund University, Lund, Sweden; Tokyo Medical and Dental University, Japan

## Abstract

The spinocerebellar systems are essential for the brain in the performance of coordinated movements, but our knowledge about the spinocerebellar interactions is very limited. Recently, several crucial pieces of information have been acquired for the spinal border cell (SBC) component of the ventral spinocerebellar tract (VSCT), as well as the effects of SBC mossy fiber activation in granule cells of the cerebellar cortex. SBCs receive monosynaptic input from the reticulospinal tract (RST), which is an important driving system under locomotion, and disynaptic inhibition from Ib muscle afferents. The patterns of activity of RST neurons and Ib afferents under locomotion are known. The activity of VSCT neurons under fictive locomotion, i.e. without sensory feedback, is also known, but there is little information on how these neurons behave under actual locomotion and for cerebellar granule cells receiving SBC input this is completely unknown. But the available information makes it possible to simulate the interactions between the spinal and cerebellar neuronal circuitries with a relatively large set of biological constraints. Using a model of the various neuronal elements and the network they compose, we simulated the modulation of the SBCs and their target granule cells under locomotion and hence generated testable predictions of their general pattern of modulation under this condition. This particular system offers a unique opportunity to simulate these interactions with a limited number of assumptions, which helps making the model biologically plausible. Similar principles of information processing may be expected to apply to all spinocerebellar systems.

## Introduction

The massive spinocerebellar systems [1], [2], which constitute a substantial part of the total mossy fiber input to the cerebellum, are presumably crucial for the cerebellum to be able to generate the necessary mossy fiber signals for fulfilling the function of the cerebellum during movements that require coordination [3]. However, there has been few papers dealing with spinocerebellar interactions, and there is little knowledge about how they could work at the neuronal circuitry level. In the present study, we take advantage of recently acquired information on the processing of inputs in a set of spinocerebellar neurons, and our own demonstration of how the cerebellar granule cells receive this input, to provide a case study of how spinocerebellar information processing may work under ongoing motor control. Our study is limited to the Spinal Border cells (SBCs), which have been sufficiently characterized to allow us to simulate their functions with relatively few assumptions.

SBCs are a separate component of the ventral spinocerebellar tract (VSCT) [4], [5]. SBCs receive a massive excitatory input from the reticulospinal tract (RST), representing a motor command, and powerful inhibition from Ib interneurons, representing a main sensory feedback to the SBCs [6]–[8]. Given the strong input from the RST, SBCs are likely to participate in the control of locomotion, similar to other cells of the VSCT [9], [10]. SBCs as a group also features input from group II and possibly group Ia afferents. However, about half of the individual SBCs do not receive group I excitatory synaptic inputs [1], [11], [12], even though they still receive inhibitory synaptic input driven by Ib afferents from combinations of synergistic muscles [1], [11], [12].

Spinocerebellar fibers terminate in the cerebellar cortex as mossy fibers, which make synaptic contacts with granule cells and, to some extent, Golgi cells. Golgi cells make inhibitory synapses with granule cells and could hence be a factor in determining the outcome of spinocerebellar processing. However, Golgi cells *in vivo* seem to have mainly a slow, modulatory role in setting the granule cell excitability [3], [13]–[15], which can be useful in differentiating granule cells activated by similar inputs [3] but not for determining the granule cell processing on the short timescale. Hence, the main point of interest for the present study is how the spinocerebellar mossy fiber information is integrated and represented at the level of the granule cells.

SBCs constitute the only spinocerebellar system that ascends in the contralateral funiculus and terminates in the sublobulus C1 of the paravermal region of the cerebellar posterior lobe [4]. As such it offers the opportunity to study the granule cell responses to inputs from this system in isolation. Although restricted to a small population of the granule cells in this region, these granule cells can display powerful responses to activation of the SBC tract [16]. In the present study, we aim to simulate the responses of SBC processing granule cells during locomotion, based on the previously described integrative properties of granule cells *in vivo* [13], [15], [17], [18]. These modeled granule cells were provided a simulated drive from SBC firing, generated from the known modulation of RST neurons [19], [20] and Ib afferents of muscles activated under locomotion [21]. To our knowledge, this paper represents the first simulation of the spinocerebellar interactions at the neuronal circuitry level, and illustrates how a single component of a large population code can contribute individually to generate signals useful for coordination tasks.

## Materials and Methods

### Data from *in vivo* recordings of granule cells in sublobule C1

To explore the responses of granule cells in the sublobulus C1 of the cerebellar posterior lobe to activation of the SBCs, and to calibrate our granule cell model, we made a systematic investigation, which is the subject of a separate paper [16]. The present paper includes a few examples from the data in that paper. For completeness, we give a brief description of the methods also here. The experiments were made in the acute decerebrated preparation of the cat. The cats were prepared as previously described [22]–[24]. *In vivo* patch clamp recordings were made from granule cells of the sublobulus C1 of the cerebellar cortex using patch pipettes pulled to 6–19 MOhm (potassium-gluconate based internal solution). In order to activate the SBC tract, the only pathway that ascends from the lumbar segments in the contralateral funiculus and terminates in sublobulus C1 [4], the contralateral lateral funiculus of the T7–T8 segments was stimulated with tungsten microelectrodes with exposed tips of 30–120 um.

### Granule cell model

The granule cells were simulated using an exponential integrate-and-fire model [25] with added stochastic component, *σ_N_dW* described by Eq. (1). *W* denotes Brownian motion of the membrane potential and the entire equation is short hand notation of the corresponding stochastic integral equation (see e.g. [26] for a more complete description).
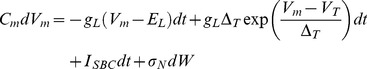
(1)where *V_m_* is the membrane potential of the model neuron. The membrane capacitance and conductance, *C_m_* and *g_L_*, and the reversal potential *E_L_* where taken from [15]. The shape and properties of the action potentials are determined by the threshold *V_T_* and the shape parameter Δ*_T_*. *V_T_* and Δ*_T_*, together with the amplitude of the noise *σ_N_* were chosen to emulate the granule cell response *in vivo* to single and triple pulse input. Whenever the membrane potential of the model reached 0 mV the model was considered to have produced an action potential, and the membrane potential was reset. The synaptic input *I_SBC_* to the granule cell models was modeled as the AMPA-receptor synapses in [27] in Eq. (2).
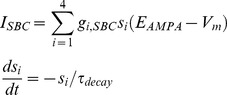
(2)where *s_i_* is the activation level of the *i^th^* SBC to granule cell synapse that is set to 1 whenever the *i^th^* SBC fires. *τ_decay_* is the time constant that determines how fast *s* returns to 0 following a spike and *E_AMPA_* is the reversal potential for the post-synaptic current. The synaptic conductances, *g_i_*_,*SBC*_ were chosen to create unitary EPSPs of the same amplitude as the EPSPs in [15] (from Fig. 6 in that paper). The parameters used to simulate the granule cells are shown in Table 1.

**Table 1:** Parameters for the neuron models.

SBC model		
*c_I_*	2.6643·10^6^ A^−1^	
Δ*_I_*	−5.8545	
*c_x_*	85.6 Hz	
Δ*_x_*	5.41	

Granule cell models		
*g_L_*	1.6 nS	(*R_M_* = 625 MΩ, *τ_M_* = 3.125 ms)
*C_m_*	5 pF	
*E_L_*	−58 mV/−57 mV	
*V_T_*	−51 mV	
Δ*_T_*	1 mV	
*σ_N_*	1.7 ·10^−6^	
*E_AMPA_*	0 mV	
*τ_decay_*	0.006 s	
*g_SBC_*	0.26 nS	

Note that the SBC model is phenomenological and that the parameters have no biophysical interpretation. The units after *c_x_* and *c_I_* are there to indicate that *x* in Eq. (3) is a dimensionless quantity.

### Spinal-border-cell model

The spike trains of the SBCs where created using an inhomogeneous point process, with an instantaneous rate related to the membrane potential of the neuron [28]. The granule cells and spinal neurons were simulated using different models of spike generation for two reasons. Firstly, VSCT neurons, of which the SBCs are a subpopulation, work as straight-forward rate coders during fictive locomotion [9] whereas granule cells differ from these and other rate coding neurons by having a great gap between the resting potential and firing threshold [15] allowing them to have fast transient responses that cannot be captured by the escape rate model but can be described by the integrate-and-fire model. Secondly, whereas the data from VSCT/SBC neurons exist in the form of firing rate modulations to relatively slowly modulated intracellular depolarizations, suitable for escape rate models such as the one we use [28], the data available for granule cells responding to SBC tract stimulation, which generates artificially synchronized and very sharp population EPSPs, is more suitable to approximate with the integrate-and-fire model.

The applicability of the used inhomogeneous point process, and its verification in *in vivo* recordings from spinal neurons, is treated in detail in a separate paper [28]. Briefly, the spike firing rate was described by a modulated log-normal hazard function that was fitted to respond with the same intensity as the VSCT neurons in [9], [10] when subjected to input that depolarizes the membrane potential in the range of 0 and 7 mV from the resting potential, similar to the maximal depolarization of 7.1 mV recorded in VSCT neurons under fictive locomotion [9].

The modulation of the intensity of the excitatory RST and inhibitory Ib input to the RST was interpolated from [19] using cubic splines to recreate the overall shape of the RST intensity and EMG activity of Srt and VL.

The lognormal distributions were modulated according to Eq. (3) [28].
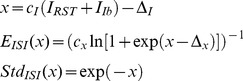
(3)where *E_ISI_* is the mean and *Std_ISI_* is the standard deviation of the lognormal distribution of the inter-spike intervals (ISI). *x* is a dimensionless parameter that translates between the input to the model and the parametric relationship between *E_ISI_* and *Std_ISI_*. *c_x_* and Δ*_x_* are parameters that govern the parametric relationship between the mean and standard deviation of the model, and *c_I_* and Δ*_I_* are parameters that govern the relationship between the input current and the modulation of the firing statistics. The synaptic input to the SBC model, *I_RST_*+*I_Ib_*, were modeled as a current created from a linear combination of excitatory input from RST and inhibitory input from inhibitory interneurons. The parameters that were used to simulate the SBC model comes from one of the spinal interneurons in [28] and can be found in Table 1.

The mean and standard deviation of a lognormal distribution can be converted to the mean *σ* and standard deviation *μ* of the underlying normal distribution according to Eq. (4) as these are commonly used to parameterize the log-normal distribution.
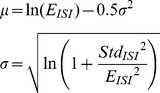
(4)
Eq. (5) is the hazard rate of the lognormal distribution that was used to simulate the inhomogeneous point process.
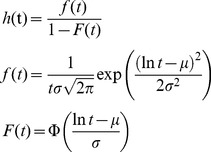
(5)where *f*(*t*) is the probability density function and *F*(*t*) the cumulative density function of the log-normal distribution and *h*(*t*) is the instantaneous hazard rate, determining the probability of a spike at time *t*. Φ denotes the cumulative density function of the standard normal distribution.

### Simulation and data analysis

The model equations were solved using a standard 4^th^ order Runge-Kutta solver with a Δ*t* = 0.1 ms timestep using custom designed software and the subsequent data analysis was performed with MATLAB (Mathworks). The same time step was used to determine whether the SBC models fired an action potential using the hazard rate from Eq. (5) multiplied with the time step, *h*(*t*)Δ*t*, as the probability of an action potential being generated.

The instantaneous firing frequency (IFF) was used as a measure of the spike response in order to facilitate comparison of the results. The time of individual spikes were binned and the IFF was calculated as the probability of a spike during that bin divided by the bin width. In cases where the firing probability is more applicable (i.e. during transient behavior) it is displayed alongside the IFF.

In order to evaluate whether a recorded behavior was predicted by the model, the 95% confidence interval of the IFF histograms of the model were created using a Monte Carlo simulation. The model was simulated to respond to the same number of traces as was recorded *in vivo*. This procedure was repeated 500 times which allowed the mean response and confidence interval to be calculated for each bin. If the *in vivo* response fell within the confidence bounds in more than 95% of the bins it was considered to predict the *in vivo* data. Note that long-latency responses were not included in the analysis.

## Results

**Figure 1:**
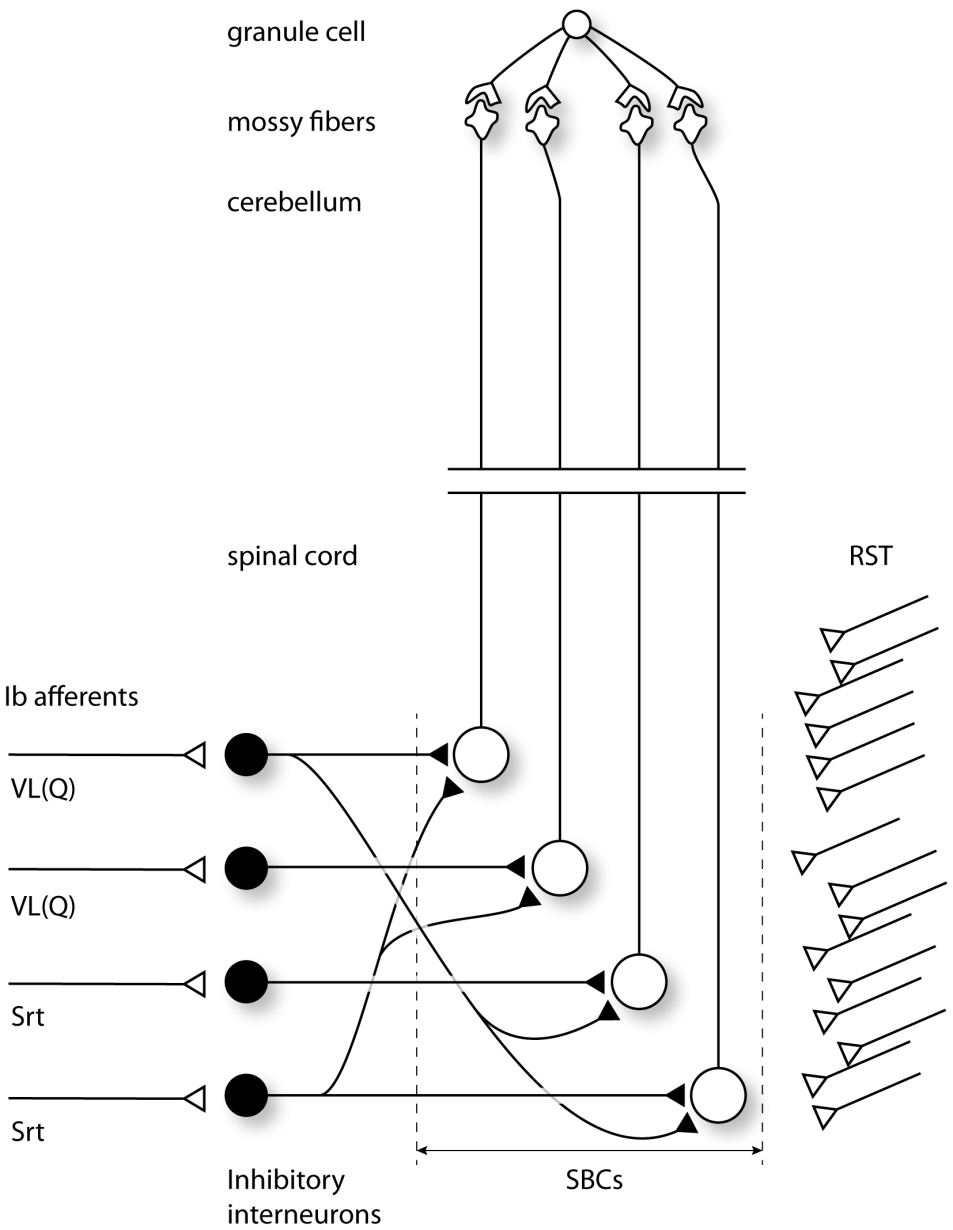
Simulated network scenario. Each granule cell was simulated to receive 4 mossy fiber inputs from the SBCs. Each simulated SBC received a massive, excitatory, monosynaptic reticulospinal input and a variable number of inhibitory interneuron inputs from the Ib interneurons driven by Ib afferents from two different hindlimb muscles, vastus lateralis (quadriceps) (VL(Q)) and Sartorius (Srt).

Figure 1 illustrates the network scenario explored in the present study. Our granule cell model could be simulated to receive any number of Spinal Border cell (SBC) mossy fibers. However, based on experimental support provided below, our paper only deals with the scenario that all four mossy fibers [15], [18], [29] are derived from the SBCs. In addition, we focused on the population of SBC neurons which receives Ib inhibitory input but do not receive group I excitation. We also limited the convergence of Ib afferent input to SBCs to the vastus lateralis/quadriceps (VL) and the sartorius (Srt) muscles, a convergence that has been demonstrated for SBCs lacking group I excitation [11].

### Granule cells

**Figure 2:**
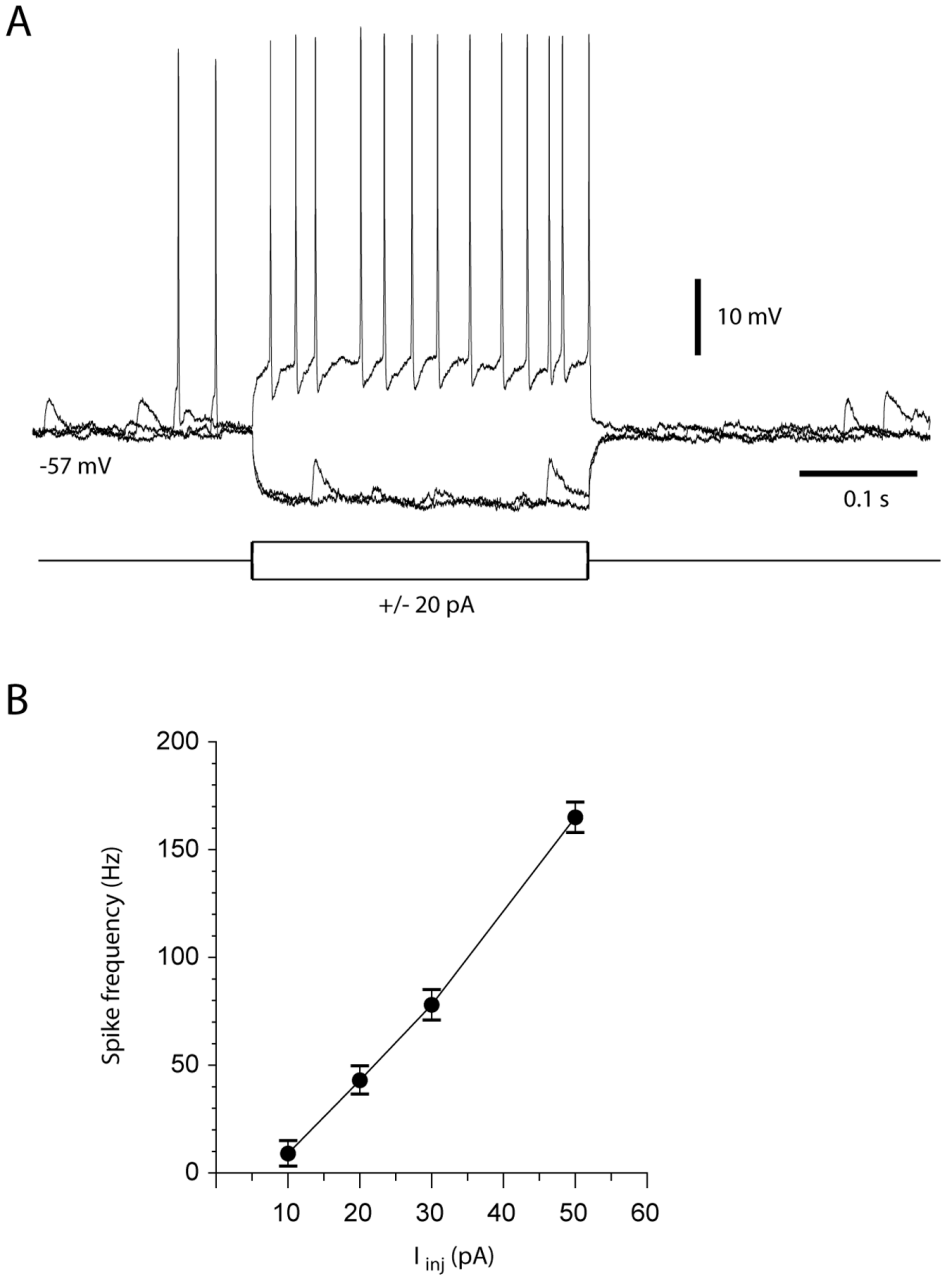
Example of *in vivo* granule cell properties in the cerebellar target region of SBC inputs. (A) Example of an intracellular granule cell recording and its responses to rectangular current steps. (B) Relationship between current injected and average firing frequency (measured from the average interspike intervals between the first and the last spikes during the current step) for the same cell as in (A). The diagram illustrates the mean and standard deviation for firing frequencies obtained at different current values (N = 10 for each current value).

The membrane properties and the mossy fiber synaptic properties of our granule cell model were based on the data presented in a previous investigation of granule cells in the anterior lobe using the *in vivo* whole cell patch clamp technique [15]. However, in order to verify that the granule cells in the region that receives SBC tract inputs behave in a similar fashion, we aimed to also make whole cell recordings from these cells in the sublobulus C1. Figure 2 presents one such recording and the relationship between the injected current and average firing frequency in this cell. We took this example as a verification that our granule cell model, based on recordings from granule cells primarily in the paravermis of the anterior lobe [15], also applied to the sublobulus C1 of the paravermis of the posterior lobe.

**Figure 3:**
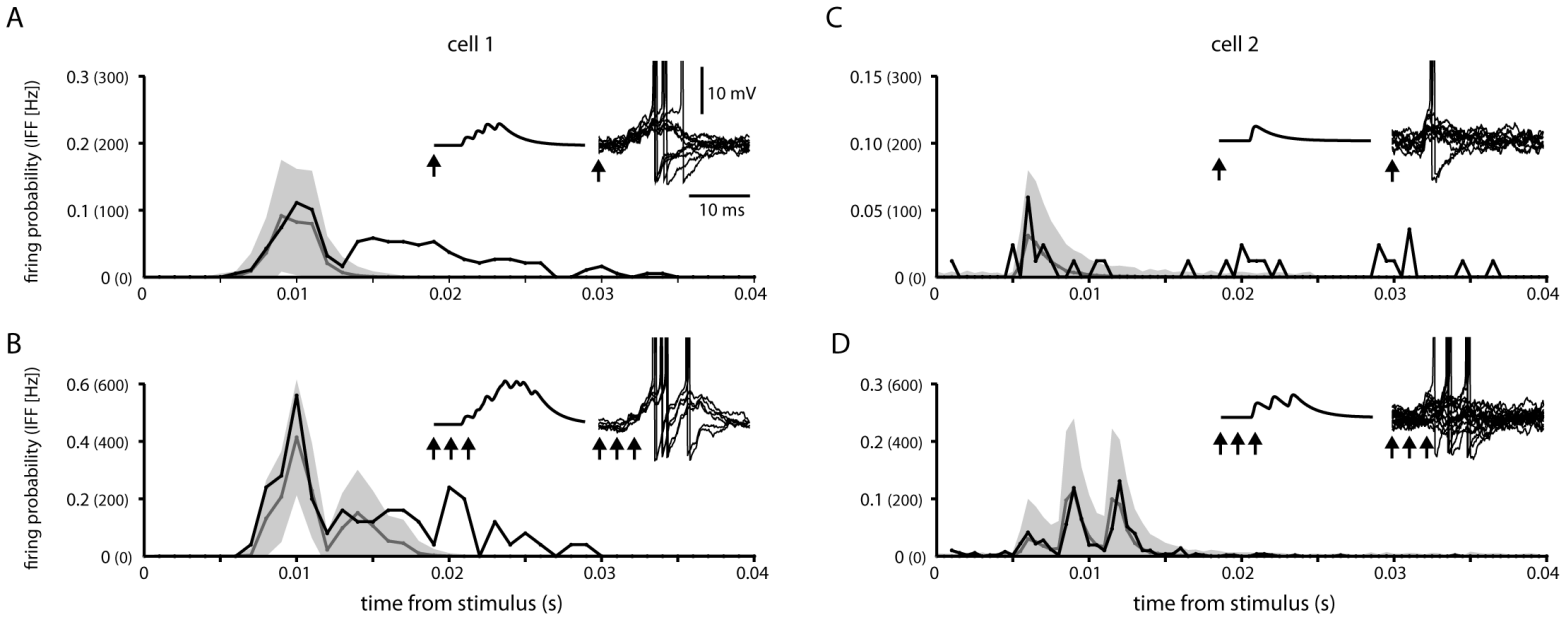
Comparison between recorded and simulated granule cell responses. (A) Histogram of the recorded granule cell response to 1 pulse stimulation to the SBC tract at 0.3 mA (black line) compared to a histogram with the average spike output of the model (grey line). The 95% confidence bound of the simulation response is shown as a grey area behind the lines. The instantaneous firing frequency (IFF) is shown for comparison with the firing probability and equals the firing probability divided by the bin width. The arrows indicate the time of stimulation both related to the synaptic input to the model (left inset), and superimposed example traces of the simulated response (right inset). (B) Same granule cell and model as in (A) but with a 3 pulse stimulus (3 ms inter-pulse intervals). (C) The response of a different granule cell, and its adapted model, to a 1 pulse stimulus of the SBC tract. Note that the models in (A) and (C) only differ with respect to the resting membrane potential of the granule cells and the latency of the EPSP input (compare left insets). (D) Same as in (C) but with a 3 pulse stimulus (3 ms inter-pulse intervals).

In order to calibrate the spike firing properties of our granule cell model, we used data obtained from recordings of sublobule C1 granule cell responses to SBC tract single and triple pulse stimulation [16] (Fig. 3). In these cases, the intensity of the response was comparable to those evoked through the cuneocerebellar and reticulocerebellar tracts in granule cells recorded in the anterior lobe, for which a more systematic analysis of the composition of the synaptic input has been made previously. At least for the anterior lobe, this previous analysis indicated that all four mossy fiber inputs to the granule cell were derived from the same input source [15], [18]. Therefore, in the present paper, we simulated only granule cells that receive SBC mossy fiber input on all four dendrites. For a granule cell in which a lower proportion of the mossy fiber synaptic inputs were driven by the SBC tract input, the synaptic input signal would display a similar profile as we present in the remainder of this paper, but the amplitude of the modulation of the membrane potential and the influence on the spike output would be lower.

Under these modeling conditions, we adapted the spike generation of two granule cells, chosen because of their different patterns of spike responses to the SBC tract stimulation [16], so that the model displayed comparable spike responses as in the *in vivo* setting. The model parameters where chosen such that the model could predict the *in vivo* responses within a 95% confidence bound (Fig. 3). Even though the *in vivo* responses to SBC tract stimulation of the two cells were distinctly different, the model could capture both response patterns solely by changing the latencies of the four individual mossy fiber synaptic inputs and the resting potential of the granule cells (−58 mV and −57 mV respectively). The fast response of the second cell (Fig. 3 C,D) could only be captured using a synchronous synaptic input from four SBCs (left inset Fig. 3C), while the more slow response of the first cell required different response latencies for the four SBC synaptic inputs (Fig. 3A,B). The scenario of different response latency times of SBC inputs is compatible with the known differences in the conduction velocities of VSCT neurons [30]. Note that later parts of the recorded granule cell responses (see for example Fig. 3A) is likely to represent input derived from long-latency synaptic activation of the SBCs via other pathways (i.e. an artifact of the experimental setup) [16] and we did not aim to capture this behavior in our model.

### Spinal Border cells

Since the lumbal spinocerebellar systems and the reticulospinal tract are strongly modulated under locomotion [10], [31], [32], as are the neurons of the paravermal cerebellar cortex [33]–[37], we wanted to design our model to explore the spinocerebellar interactions in the cat during this behavioural context. Therefore, the SBCs were simulated to receive excitatory drive from the RST and inhibitory input from spinal interneurons, which were simulated to receive only primary afferent input of the Ib muscle afferent type.

SBCs are innervated by a massive (approximately 5000) number of excitatory synaptic inputs from the RST and a comparable number of synaptic inputs from inhibitory interneurons [7], [8]. At the population level, these synaptic inputs can generate a total depolarization of up to 5 mV and a total hyperpolarization of about 7 mV when activated synchronously, using electrical stimulation of the descending tracts or afferent nerves [7], [8], [11], [38], [39]. It follows, similar to the magnitude of synaptic weights in other synaptic junctions of the cat spinal cord [40], that the responses of the individual synaptic connections are likely to amount to only a few µVs in amplitude in SBCs. On the other hand, since the SBCs receive so many RST synapses and inhibitory synapses from interneurons activated by Ib afferents, concerted activation of these inputs will be translated to relatively massive excitatory and inhibitory modulations of the intracellular potential (shown for VSCT neurons under fictive locomotion [9]), which are much slower than the responses obtained by synchronous fiber activation obtained on tract stimulation and nerve shock stimulation [7], [8]. The amplitude of these intracellular membrane potential changes should be related to the average firing rate of the neurons that converge onto the individual SBC. In our simulation of the intracellular signals of the SBCs, these smooth total synaptic input signals are reduced to a single input current that correspond to the average, summed activity of the populations of excitatory and inhibitory neurons that innervate the SBC.

**Figure 4:**
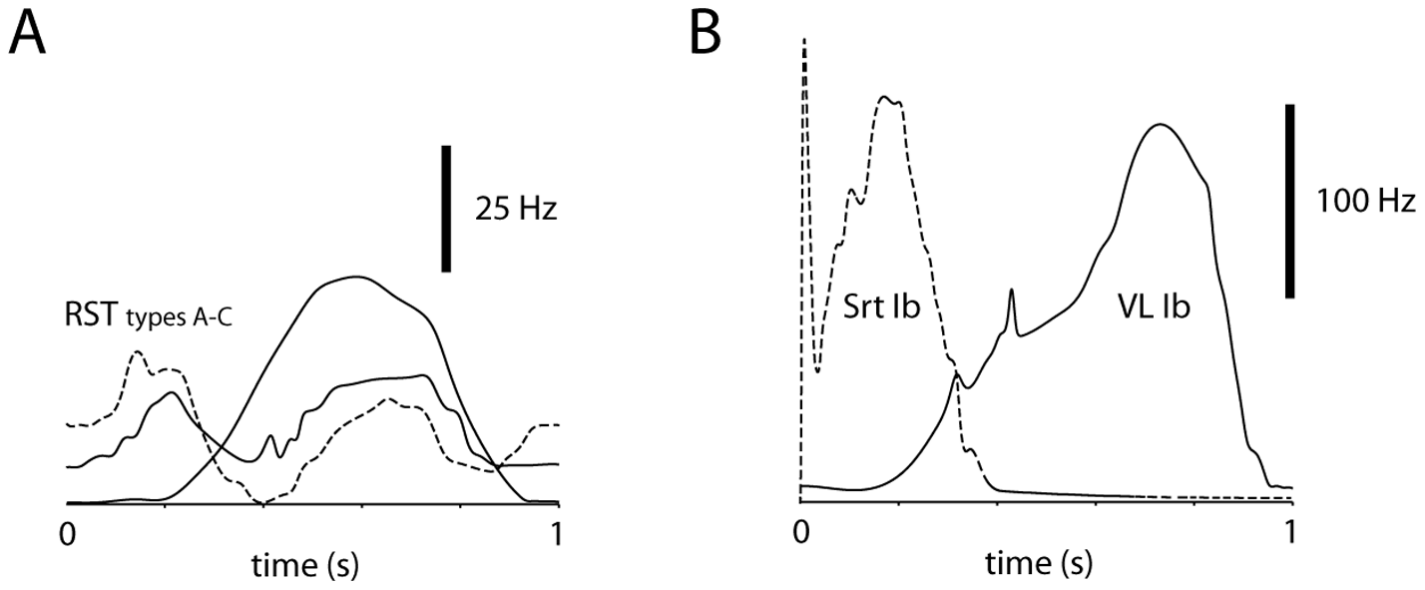
Time-courses of simulated synaptic inputs. The simulated synaptic inputs were adapted from data recorded under locomotion [19]–[21]. (A) Examples of RST neuron activity under locomotion. These time-courses were summated and represented the excitatory synaptic drive of the SBCs. (B) Examples of EMG/Ib afferent activity of the two muscles simulated to provide inhibitory synaptic inputs to the SBCs. These inputs were weighted in a variable fashion in the simulated SBCs, and the inhibitory input was linearly summated with the excitatory RST input.

The time-course of the RST excitatory modulation was adapted from [19], which illustrate three examples of RST neuron modulation during locomotion (Fig. 4A). We adapted functions to reproduce the individual time-courses of RST neuron activation (see Methods) and then summed these functions to generate an average RST neuron activity used to drive the excitatory input to the simulated SBCs. The simulated Ib afferent activity was similarly taken directly from EMG recordings of the specific muscles of interest obtained during locomotion [19]. This was motivated by a study that has shown that during locomotion the modulation of an ensemble of Ib afferents essentially mirrors the EMG activity of the corresponding muscle [21]. An essential assumption of the present model is that the Ib afferent activity is transmitted linearly via the spinal interneurons to the SBCs – consequently, the simulated Ib afferent activity was simply provided with a negative synaptic weight, with variable gain as shown below, to the SBCs. We focused on Ib afferent input from the vastus lateralis (quadriceps) muscle and the sartorius muscle (Fig. 4B), whose Ib afferents were given different relative weights in the simulated SBCs to explore the effects of varying the convergence pattern in the SBC/spinal interneurons.

**Figure 5:**
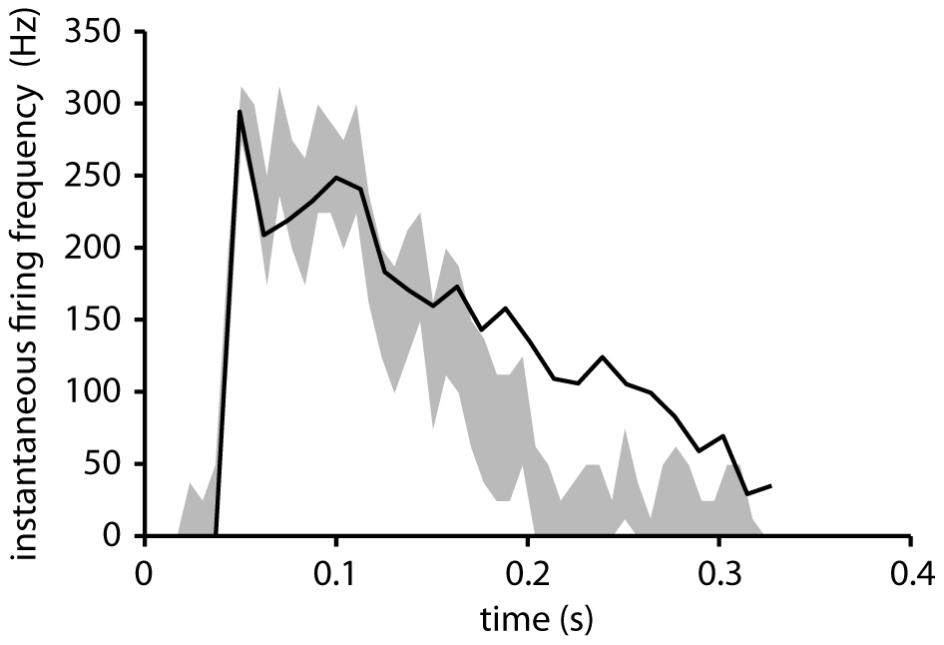
SBC model prediction compared to actual firing recorded in a VSCT neuron. The spike response of the SBC model to an input intracellular signal recorded during one step cycle (adapted from [9]). The gray area indicates the 95% confidence bounds of the model response and the solid line the average measured instantaneous firing frequency from 12 cycles [9]. Note that while the model does not reproduce the measured response beyond 0.2 s, it captures the initial transient from 0 to approximately 300 Hz, and also the subsequent slow decay of the firing frequency. Since the input to the model and the firing frequency are naturally not from the same step cycle, perfect overlap cannot be expected.

In order to validate that the modelled SBCs could display similar input-output relationships as actual SBCs, we provided our SBC model with the intracellular signal recorded from a VSCT neuron during a step-cycle and compared the spike output with that recorded from the same neuron (recorded data taken from figure panels in Ref. [9]) (Fig. 5). The model captured both the initial steep rise in firing frequency and its slow decay following the initial peak (Fig. 5). 200 ms into the step, the reproduction was poorer, but the discrepancy can be explained by that the recorded intracellular signals differed from step to step and the recorded firing displayed was an average of 12 step cycles.

### Spinocerebellar interactions

**Figure 6:**
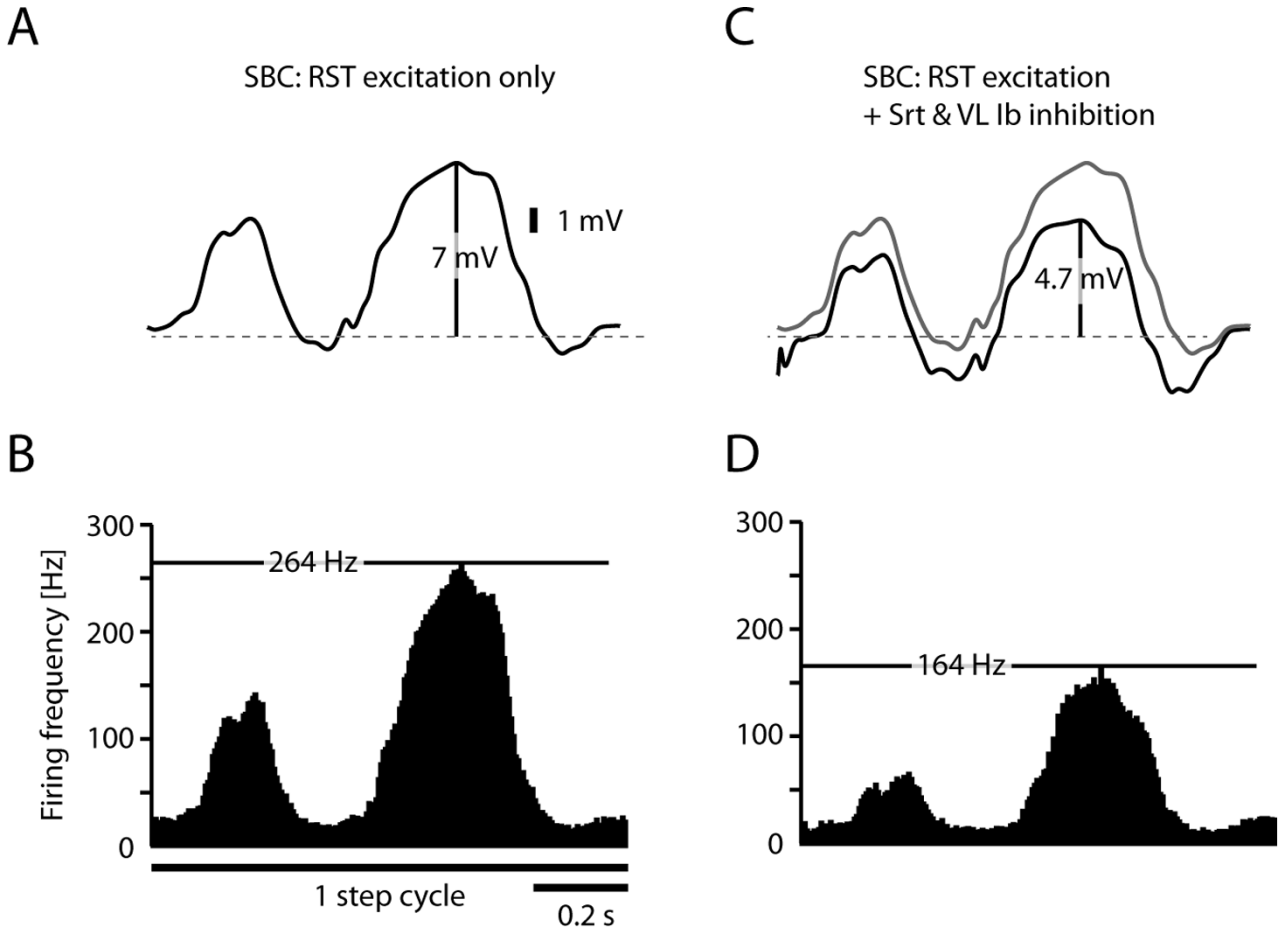
Simulated activity of SBCs receiving RST input and Ib inhibitory input. (A) Simulated intracellular SBC signal of RST excitation only during one step cycle. The amplitude of the intracellular signal in the model (7 mV) corresponds to that recorded in VSCT neurons under fictive locomotion in paralyzed cats (7.1 mV) [9]. (B) Corresponding simulated spike responses in SBCs, summarized in histograms with the instantaneous firing rate (5 ms bin width) for the full step cycle. The maximum firing rate of the model is indicated by the horizontal black line (264 Hz). (C) Same simulation as in (A), but in this case including inhibitory synaptic inputs from the Srt and VL Ib inhibitory interneuron input (black line, compare with the response without inhibition, illustrated by the grey line). (D) Spike responses for the SBC neuron with summation of RST excitation with the Ib afferent evoked inhibition.

The result of the SBC simulation can be seen in Figure 6. The amplitude of the simulated intracellular signal (7.0 mV), displayed without action potentials (Fig. 6A), corresponded to that recorded in VSCT neurons during fictive locomotion (7.1 mV) [9], i.e. without Ib inhibition as the muscle nerves were cut. It generated a spike output (with a peak amplitude of 264 Hz) in the modeled SBC neuron which was comparable to that recorded in VSCT neurons during actual and fictive locomotion (peak amplitude of 100–300 Hz) [9], [10]. Figure 6B illustrates the average instantaneous SBC firing rate across 1000 step cycles. When the simulated SBC neurons were provided with inhibitory input driven by simulated Ib afferent activity, the intracellular signal and the firing rate were modulated accordingly (Fig. 6C,D).

**Figure 7:**
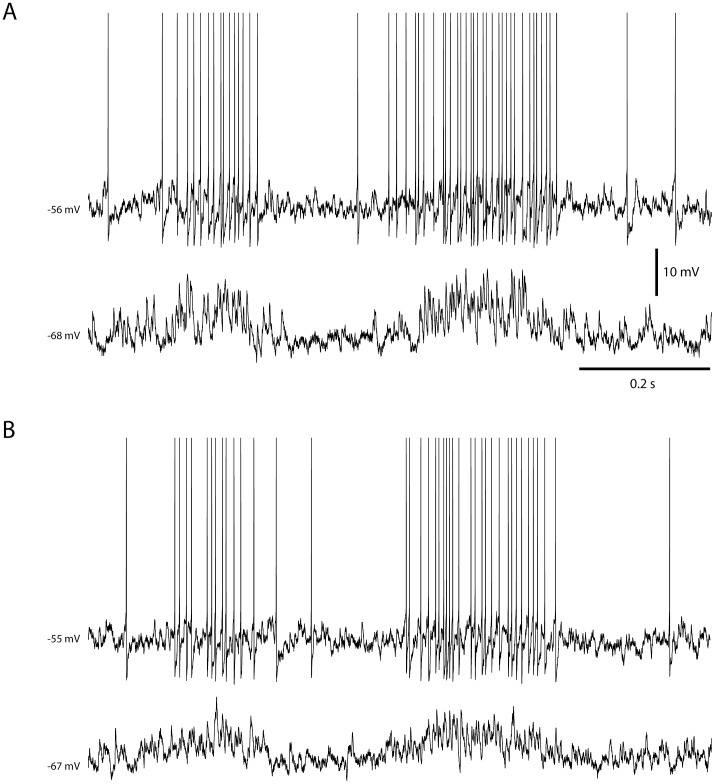
Examples of simulated granule cell responses during one step cycle. Panels (A) and (B) represent simulations for granule cell 1 and granule cell 2, respectively (see Fig. 3). In both panels, the top trace illustrates the response of the simulated granule cell simulated with a normal membrane potential. The lower trace illustrates the responses of the stimulated granule cell with −20 pA bias current, preventing the cell from generating spikes.

Figure 7 illustrates the intracellular membrane potential and spike responses of the two simulated granule cells that each received inputs from four simulated SBCs during one locomotion step cycle. Fig. 6A illustrates the simulated response of the granule cell simulated in Fig. 3A,B (cell #1) whereas Fig. 6B illustrates the granule cell simulated in Fig. 3C,D (cell #2). In the model, the granule cell simply integrates the EPSPs evoked by spikes in the individual SBCs (Fig. 6B) and the Ib inhibition in the SBCs (Fig. 6D) is expressed as a reduction in the number of EPSPs generated in the granule cell.

**Figure 8:**
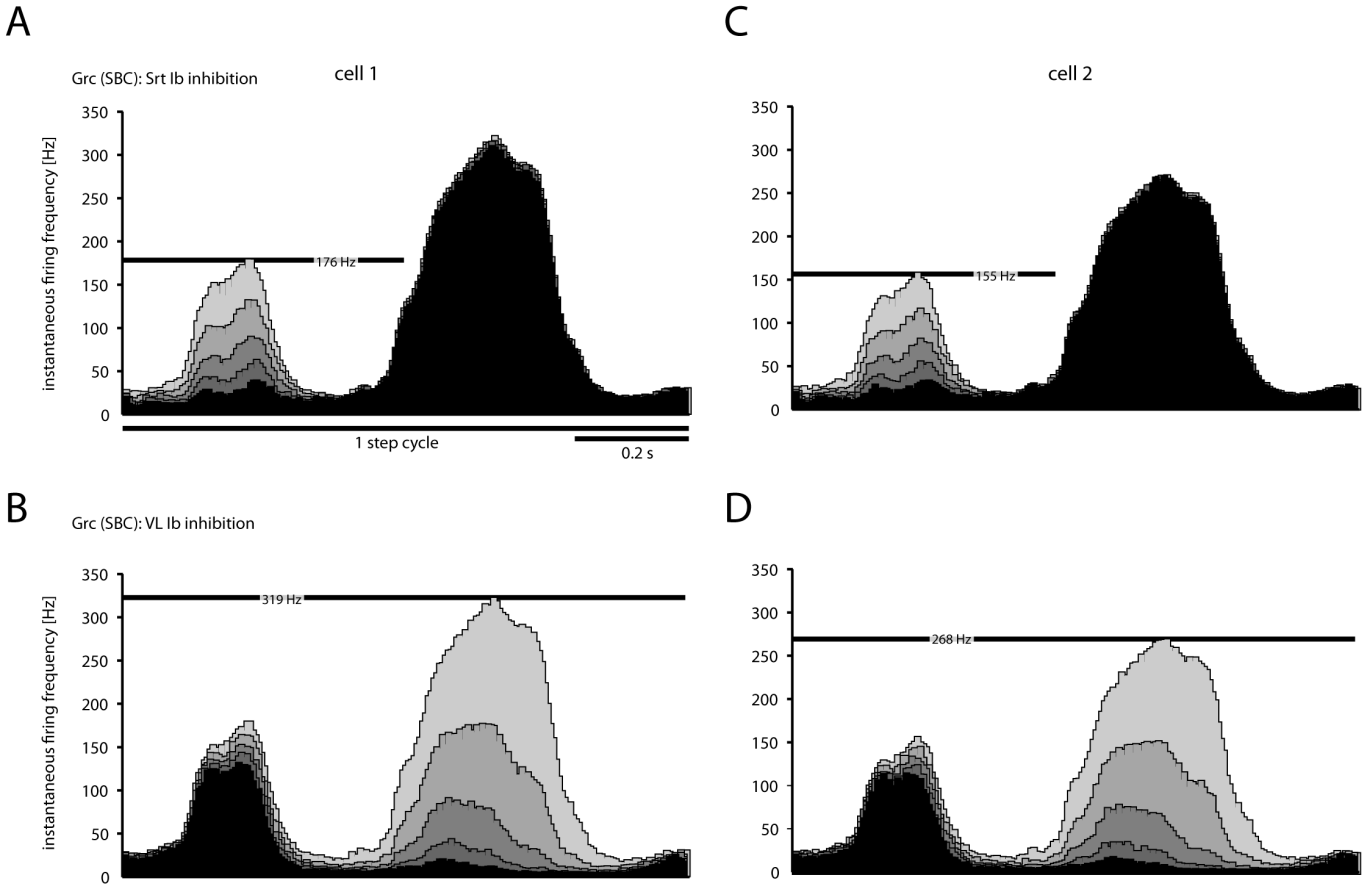
Effect on granule cells spike responses of grading the synaptic weights at the level of the SBCs of the Ib inhibitory interneurons from different muscles. (A) Histogram with the instantaneous firing rate (5 ms bin width) of the model response, using the model that reproduced the response of granule cell 1 (see Fig. 3). In this case, all SBCs were simulated to receive Ib inhibition from the Srt muscle only. The weight of the inhibitory synapses between the Ib inhibitory interneurons were varied in five steps, 0.2–1.0 in arbitrary values. The effect of the different weights on the pattern and level of granule cell firing is shown in the histogram as bars in different shades of grey. (B) Similar display as in A, but in this case the synaptic weights of the Ib interneurons activated by the VL muscle were varied between 0.2–1.0, and the Srt weights were set to 0. Panels (C) and (D) are the same as (A) and (B), but using the model that reproduced the response of granule cell 2.

We next used the modeled network to explore the effect of varying the synaptic weights with which the Ib inhibitory inputs modulated the SBC neurons. Figure 8 illustrates how the granule cell activity varied across different levels of VL and Sart Ib afferent inhibitory synaptic weights on SBCs. As the inputs were linearly combined in the SBCs, increasing inhibitory synaptic weights resulted in linear reductions in the granule cell spiking activity corresponding to the specific temporal profiles of activation of the respective muscle. We made this simulation both for granule cell #1 (Fig. 7A,B) and cell #2 (Fig. 7C,D). Note that the two granule cells, with different responses to SBC tract stimulation (see Fig. 3) still fired with a similar temporal topography and overall firing frequency.

## Discussion

The present modeling study illustrates how SBCs and granule cells together can integrate sensorimotor information during a motor control scenario. As both granule cells and spinal neurons are described as linear integrators [17], [41] (see also extensive discussion in [3]), the integrative functions of the individual neurons are limited. However, because the spinal neurons are located in a circuitry that is an integral part of the circuitry shaping the motor command [42], [43], the spinocerebellar system can provide the population of granule cells with a more integrated, functionally relevant set of inputs than the raw sensory signals or efferent commands could provide separately.

Generally, rather than viewing the cerebellum as a separate functional structure, its functions is intimately related to the structure from which it receives its inputs. In the case of the spinal cord, the cerebellum, through the spinocerebellar systems, can expand the sensorimotor capabilities the spinal circuitry provides by allowing for approximations of more complex functions that describe interactions across or within limb segments [3]. This could for example correspond to the interactions between synergistic muscles activated during learnt movement patterns such as locomotion. In the present paper we focused on the simplest possible instantiation of the spinocerebellar network described in the literature, i.e. the integration of excitatory RST inputs with inhibitory inputs generated by Ib afferents via spinal interneurons in SBCs. However, the general functional principles are likely to be the same for all spinocerebellar systems, although more complex spinal processing may be superimposed on these principles.

### Main limitations of out model

The main potential limitations of the present study lie in the generation of spike output in the SBCs and in its afferent inhibitory interneurons. However, spinal interneurons and spinocerebellar neurons appear to be essentially linear integrators [28], [41], [44]–[49] and the details of how the spikes are generated may matter little in a system that seems to operate primarily according to the rate coding principle. The convergence pattern of Ib afferents from different muscles in the inhibitory interneurons are more extensive than in our simulation [11] but the effects of possible additional inhibitory synaptic inputs from other muscles are illustrated in principle in Fig. 7. Basically, adding inhibitory effects from other muscles is equal to adjusting their synaptic weights, and similar to the effects of varying the amount of inhibition in Fig. 7 the effect of other muscles will be linearly added.

### Effects of more complex networks in the spinal circuitry

Under the conditions tested here, the granule cells responded to a linear combination of the Ib afferent sensory input and the RST motor command. While such an organization is likely for the descending RST signals that directly excite the SBCs, the sensory inhibition is mediated through at least one additional synaptic relay, i.e. the Ib inhibitory interneuron, which may conceal additional complexity. These interneurons may not be limited to mediating Ib input, but may in many cases mediate a combination of input from group Ia, group Ib and group II muscle afferents [1], [11], [12], the descending motor command [40], and also other local interneurons. Hence, the SBCs would via these interneurons be able to sample the state of the spinal cord and thereby providing a higher level of representation of the local sensorimotor information to the cerebellum [43]. The additional functions that could be represented in the system due to any complexity in how the Ib interneurons integrate information is beyond the scope of the present paper since it would in that case be a property of the interneurons. But these functions would be expected to be linearly combined with the functions we explored in the present paper, due to the linearity of spinal interneurons [16].

### Functional interpretations

The spinal neuronal network described here illustrates the formation of individual projections through a multi-dimensional sensorimotor space, the theoretical advantages of which were explored in [3] and for which there is direct experimental support [41]. In the present paper, efferent signals from the RST, which likely represent a set of motor dimensions, were recombined with Ib afferent signals from a pair of muscles (each representing a single sensor dimension). By varying the strength of the Ib inhibition from one of the muscles, the direction of the projection in these input dimensions will change. The synaptic weights by which the Ib afferents actually influence the SBC neuron presumably represent a learnt pattern, which is particularly useful to the spinocerebellar system [3]. Although we do not presently know how this learning would work at the level of the spinal cord (such learning has been demonstrated during development for cutaneous inputs to spinal neurons and withdrawal reflexes [50], [51]), in the present study we explored a range of scenarios for how different weights in this particular synaptic junction would influence the spinocerebellar processing (Fig. 7). If the signals that converge in the SBCs are functionally related, which could emerge as a result of developmental learning and is a main assumption of the present model, it offers the advantage of supplying the granule cells with relevant recombined signals [3] rather than a complete random arrangement, where each granule cell would sample a random set of input signals (i.e. the scenario portrayed in the popular models of granule layer processing originating from Marr [52]). Without this functionally selective convergence upon the granule cells, there would be a large number of granule cells that received functionally disparate input that would have no functionally relevant, non-linear interactions during normal behavior and would therefore be of limited use to the cerebellum [3].

### What happens with the information after the granule cells?

After the information is generated at the level of the granule cells, it is transmitted to the output cells of the cerebellar cortex, i.e. the Purkinje cells as well as the inhibitory interneurons innervating the Purkinje cells. Here, the information can be integrated with other sources of information, such as sensory input from the dorsal column nuclei [15], [18] and neocortical input via the pontine nuclei. How the information in this integration is weighted is probably to a large extent determined by plasticity processes, which allows both Purkinje cells and interneurons to pick out and weigh the specific pieces of information that is required for fulfilling their task of regulating the cerebellar output [3], [53], [54]. How the divergent deep cerebellar nuclear output [55], [56] subsequently is integrated with and contributing to the synergy control of the motor systems of the brain [42] remains one of the greatest challenges to the field of motor control neuroscience.

## Acknowledgments

The authors wish to thank prof. Elzbieta Jankowska for invaluable input to the theoretical design of the model.
